# The Nature and Faculties of the Sympathetic Nerve

**Published:** 1847-07-01

**Authors:** 


### THE NATURE AND FACULTIES OF THE SYMPATHETIC NERVE. By *Joseph Swan*. 8vo. pp. 55. London, 1847.

IN a former Number of this Review, we called the attention of our readers to a Pamphlet on the Nerves of the Uterus, by Mr. Swan—(Med.-Chir. Rev., April 1846.) In the present and somewhat larger publication, the author takes a more general view of the anatomy and physiology of the ganglionic nervous system; and, in connexion with it, of the par vagum.

It need scarcely be remarked that the sympathetic nerve is that part of the nervous system concerning which, both as respects its anatomy and physiology, the greatest uncertainty prevails. In late years, however, much light has been thrown upon this subject; or rather it may be said on the anatomical structure; for although there has been no lack of theories, it can scarcely be affirmed that any positive and unquestioned fact has been established with respect to the peculiar functions of the ganglia and their nerves. The following are the principal points which may be regarded as proved :—1. That in its ganglions the sympathetic offers the same elementary structures, especially gray corpuscles or vesicles, which are considered to constitute, in the case of the brain and spinal cord, an active nervous centre ; 2, that it contains fibres proper to itself, the so-called gelatinous, gray, or organic nervous filaments, originating from, or at all events connected with, the above-named vesicles ; 3, that it receives into its system tubular fibres from the cerebro-spinal system, these being derived from both sentient and motor nerves, as is seen in the case of the scattered cephalic ganglia, and also in that of the common spinal nerves where tubules come off from both the anterior and the posterior roots ; 4, That these two classes of fibres, the gelatinous and the tubular, however much they may appear to be mixed up and confused together, constitute no exception to the one great law of the nervous system—the non-intercommunication of the primitive fibrillæ, that is to say, whether traced in the interior of the ganglia or in the branches, they are always observed to maintain an independent course ; 5, that the various branches and nerves belonging to the sympathetic contain the gray and tubular fibrils in different but determinate numbers.

These are the anatomical facts, and of course they offer much suggestive evidence as to the physiological actions, upon which moreover there are some few positive but subordinate facts that have been ascertained, either by observation or by experiment. Thus it is certain that, although ordinarily the organs supplied by the sympathetic perform their functions without any known participation therein by the brain, yet occasionally impressions do traverse to and fro, sensation being excited, as for example, from irritation of the intestinal mucous membrane, and emotion, not volition, affecting the heart and other motorial organs. It is also further ascertained that impressions are mutually transmitted between the sympathetic and the true spinal system ; if, for example, in a recently decapitated kitten, the spinal cord be irritated in the neck, the heart's action is immediately quickened ; and, again, if irritation of an organ exclusively supplied by the sympathetic, as the intestine, be morbidly excited, convulsions may be induced. These few facts comprise almost all that is positively ascertained ; and it is clear that, relating to the mutual connexions of the ganglionic and cerebro-spinal system, they throw little or no light on the special or true functions of the ganglionic system.

Mr. Swan's brief exposition consists of an interesting sketch of the structure of the sympathetic, as ascertained by the aid of comparative anatomy and of its physiology. The following passage will convey to the reader the general views entertained by the author. " The great object of the sympathetic nerve is to furnish the parts it supplies with an appropriate nervous excitement of such a quality as will insure their functions without disturbing any other portion of the nervous system. It connects in different degrees all the parts of the nervous system as an harmonious whole, but brings them in so slight a degree in communion with the sensory as to allow only a perceptibility that can appreciate and respond to impulses without permitting them to proceed beyond the viscera. By preventing sensation, it becomes favourable to the production of involuntary motion, so that impulses on the lining membrane of the viscera, when sufficiently strong, are responded to, and the contraction of the muscular coat takes place. For these purposes it has a peculiar conformation, which differs more or less from the other parts of the nervous system. Although it communicates with several cerebral nerves, it assimilates most to the fifth and spinal nerves. From these and their centres it probably derives some of their essential or diffusive influence for fortifying its vital powers, but admits only just as much of their faculties as corresponds with the functions of the parts it supplies."—P. 4.

The comparative anatomy discloses some interesting facts. In the first place the plan of distribution, making allowance for diversity in the form of the body, " preserves a very great similarity, because it corresponds almost entirely with the fifth and spinal nerves;" the general principle is that, there is one ganglion to each pair of spinal nerves, but these may either remain distinct from each other, as in the cervical region of birds, where there is a small ganglion attached to each cervical nerve; or they may be massed together, as in the superior ganglion of the neck in man, which comprises in reality several distinct ganglia. The presence of large and active extremities makes it larger, especially its ganglia. Then as to texture, in the higher animals, the ganglia are close and fleshy, whilst in other classes, as in many reptiles, they are thready or plexiform; and Mr. Swan believes that the powers and excitability of the latter are inferior to those of the former.

As regards the " innate faculties" of the sympathetic, as Mr. Swan terms them, he conceives, that there are two distinct powers—" a diffusive influence, and a perceptive and motive faculty;" the former, " which forms the element of the material portion of the nervous system, the sympathetic has the power of deriving from, and through, the cerebral and spinal nerves. It can have its powers depressed or exalted; they are depressed when those of the brain and spinal marrow are low, and especially when the spinal marrow has been permanently injured; they may be exalted in any increased vital process, whether sound or morbid." As to " the perceptive and motive faculties, they are concentrated in, and have their specific powers limited to, any particular region by a ganglion or plexus;" the quality of the perceptive faculty likewise depends on the structure of the ganglia, although its powers are capable of being further exalted or depressed by the state of the diffusive influence."—*L. c.*, pp. 17, 18.

As it would exceed our limits to enter into the details of this inquiry, we shall merely extract the summary which the author has drawn up of his views.

" The sympathetic nerve has several properties peculiar to itself, and some resembling the faculties of other nerves. It is, to a certain extent, an independent system, and is a source of peculiar power. It derives its general diffusive influence, or nervous element, from the other parts of the nervous system its branches communicate with. It has appropriate faculties, and is perceptive, and not sensitive, except in pain. Its ganglia have a power according to their structure, and a capability of receiving and answering impulses. It excites involuntary motion primarily. If the impulses are strong, but still amount only to a perception, the ganglia can transmit them to the spinal marrow, and thence directly to the voluntary muscles; but if the impulses be stronger, and amount to pain, they may pass from the spinal marrow to the brain, and excite voluntary motion; or they may pass from the ganglia to the stomachic plexuses, and from these by the par vagum to the oblong medulla, and thence to the spinal marrow and its nerves, and produce vomiting. It, and especially its ganglia, have a peculiar circulation of blood by minute vessels, derived from contiguous parts, which is quiet and gentle, and increased by the activity of the organs its branches supply; it may also be increased by excitement communicated through the nervous influence, or tracts, or nerves, from the brain, and other centres, the intellect, passions, and external senses. It presides over, and connects in action the whole arterial system, particularly in the brain, spinal marrow, and nervous system generally. It belongs principally to the heart itself, but through connections with branches of the par vagum, combines the nerves of this organ with those of the lungs, and those of the muscles of the chest. It combines the abdominal viscera, and these with the abdominal muscles. It animates and combines the reproductive organs; also the urinary organs, and connects these with sentient and voluntary nerves. It combines various other nerves and parts, through minute and intricate branches. It excites the organs it supplies, to separate from the blood and food such things as are prejudicial, whilst the absorbents retain the beneficial. As its ganglia have so much power over the circulation, they, with other nerves and processes, tend to the production of heat; they, also, through a somewhat similar influence, to that in the heart, tend to the persistence of action and want of weariness, even in the voluntary muscles of birds. It determines the effect that local injuries shall excite in the heart and blood-vessels, and the extent and power of constitutional irritation for their reparation." P. 47.

We must leave our readers to form their own judgment of the value of these conclusions; only observing for ourselves that, whilst concurring in several of them, which cannot however lay claim to much novelty, from others we entirely withhold our assent.

In connexion with this inquiry, we may state that Mr. Beck, in a late number of *The Lancet,* contends that the sympathetic system is distinct from, and independent of, the cerebro-spinal system; each having its proper anatomical structure, its distinct physiological acts, and being subject to its distinct diseases. In his former paper (see Med.-Chir. Rev., Oct. 1846) he endeavoured to demonstrate the perfect distinctness of the two systems in origin, course, and distribution; and the principal object of the present contribution is to shew by the effects of ether on the uterus, that whereas the influence of this agent is upon the cerebro-spinal system, and that as the reflex functions of the spinal marrow are in abeyance, whilst the action of the uterus continues unimpaired, the inference is, that this organ must depend on some other influence than that of the cerebro-spinal system, on that namely of the sympathetic, which mainly supplies the uterus with nerves.

